# GSEPD: a Bioconductor package for RNA-seq gene set enrichment and projection display

**DOI:** 10.1186/s12859-019-2697-5

**Published:** 2019-03-06

**Authors:** Karl Stamm, Aoy Tomita-Mitchell, Serdar Bozdag

**Affiliations:** 1 Department of Mathematics, Statistics and Computer Science, Marquette University, Milwaukee, WI USA; 2 Department of Surgery, Medical College of Wisconsin, Milwaukee, WI USA

**Keywords:** RNA-Seq, Transcriptome, Gene ontology, Differential gene expression, Clustering, Visualization, Bioconductor

## Abstract

**Background:**

RNA-seq, wherein RNA transcripts expressed in a sample are sequenced and quantified, has become a widely used technique to study disease and development. With RNA-seq, transcription abundance can be measured, differential expression genes between groups and functional enrichment of those genes can be computed. However, biological insights from RNA-seq are often limited by computational analysis and the enormous volume of resulting data, preventing facile and meaningful review and interpretation of gene expression profiles. Particularly, in cases where the samples under study exhibit uncontrolled variation, deeper analysis of functional enrichment would be necessary to visualize samples’ gene expression activity under each biological function.

**Results:**

We developed a Bioconductor package rgsepd that streamlines RNA-seq data analysis by wrapping commonly used tools DESeq2 and GOSeq in a user-friendly interface and performs a gene-subset linear projection to cluster heterogeneous samples by Gene Ontology (GO) terms. Rgsepd computes significantly enriched GO terms for each experimental condition and generates multidimensional projection plots highlighting how each predefined gene set’s multidimensional expression may delineate samples.

**Conclusions:**

The rgsepd serves to automate differential expression, functional annotation, and exploratory data analyses to highlight subtle expression differences among samples based on each significant biological function.

## Background

RNA-seq is a revolutionary technology to measure genome-wide gene expression of biological samples at high resolution by sequencing messenger RNA (mRNA) molecules [1]. Common usages of RNA-Seq technology are computing transcription abundances [2], finding differentially expressed genes between two or more groups [3], de novo transcriptome assembly [4, 5] and finding novel genes and splicing patterns [6]. Among these usages, differential gene expression (DGE) analysis followed by functional enrichment is a common workflow in gene expression studies [2, 7, 8].

After RNA-seq reads are generated using a sequencing instrument, gene expression abundance is estimated by mapping the sequencing reads to a reference genome if there is an available reference genome or by building a transcriptome assembly de novo [9, 10]. DGE analysis is performed to compute statistically significant differentially expressed (DE) genes using tools such as DESeq2 [3], edgeR [11], limma [12] and Cufflinks [2]. DGE analysis could result thousands of genes, thus to better characterize the underlying biological functions of the DE genes, functional enrichment analysis is performed using tools such as GOSeq [8] and SeqGSEA [13].

However, particularly when biological samples are not well separated (e.g., mammalian tissue or human disease samples are often heterogeneous or heterocellular), a direct two-group DGE analysis can result in unmanageable lists of DE genes with uncertain significance [14]. Furthermore, batch effects may obscure the experimental signal or sample mishandling may generate outliers that perturb the experimental signal in ways unnoticed by the investigator.

In these scenarios, list of DE genes and even significantly enriched biological processes would be hard to interpret for biologists. Alternatively, after computing significantly enriched biological processes, samples could be visualized based on their activity for each of these biological processes. Per biological process visualization would enable biologists to have a deeper understanding of the samples’ activity with respect to each significant biological process.

To streamline the analysis of RNA-seq datasets to achieve the aforementioned goals, we developed a software toolkit GSEPD (gene set enrichment and projection display). GSEPD produces DE gene lists, significantly enriched gene ontology (GO) terms, and importantly their cross-product: a mapping of which genes are perturbed within each GO term, and how genes associated with those terms define the samples’ expression profiles in the context of the other RNA-Seq samples. GSEPD provides various plots and tables to summarize the results and give its users a comprehensive outlook of the underlying RNA-seq data.

We demonstrated the usage of GSEPD on a time series dataset of H1ESC cell lines [15]. GSEPD computed DE genes and significantly enriched GO terms between two time points, and clustered samples from all time points based on their activity in each significant GO term.

GSEPD is implemented as a Bioconductor package named rgsepd and freely available under GPL-3 license.

### Implementation

**Fig. 1:**
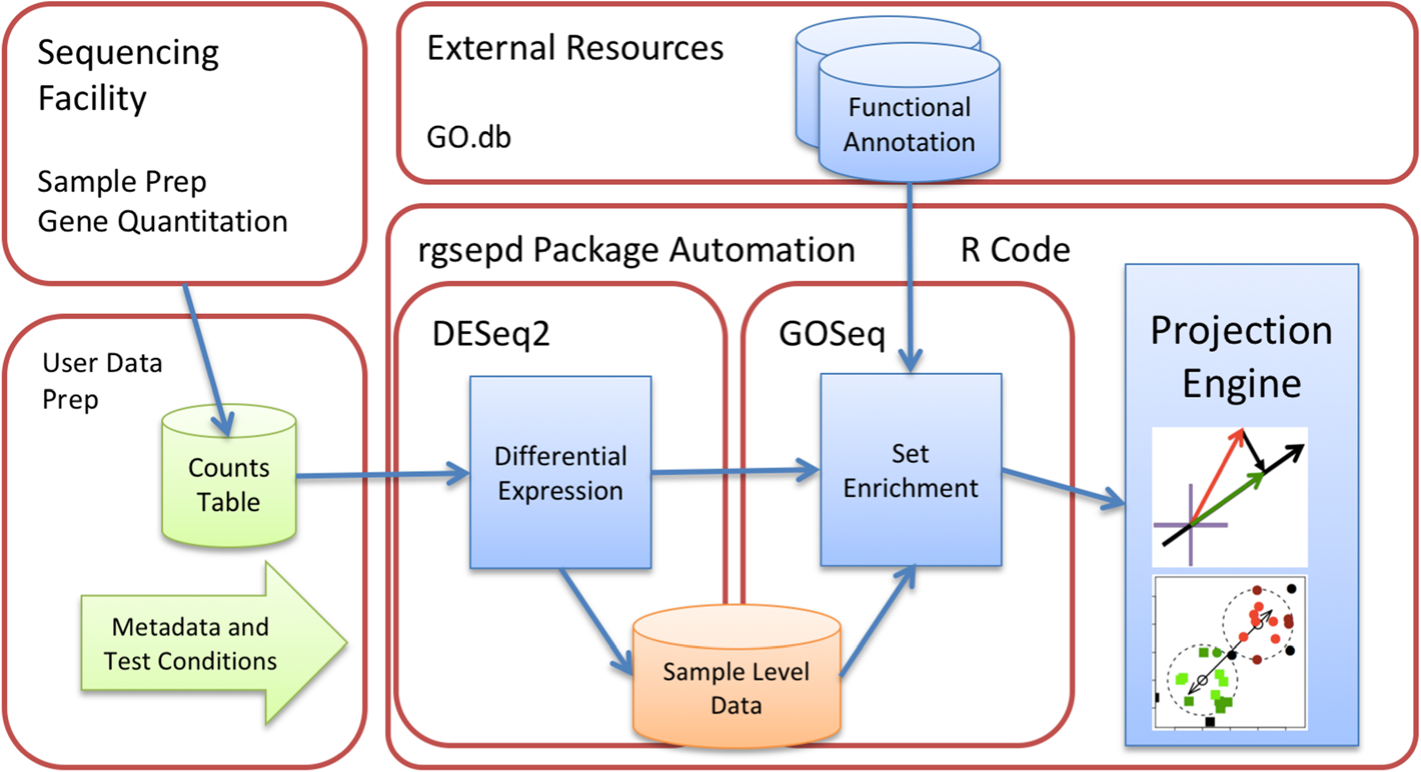
Systems Architecture diagram of the components of the GSEPD system, with major sections in red outlines. Blue items indicate automated systems. An experiment starts at the upper left, with the Sequencing Facility where the tissue samples are converted to gene expression quantification through sequencing and processing external to GSEPD. The user then creates a table of count data and defines the sample metadata and conditions to be compared (lower left, green items indicate user inputs). Across the top are External Resources, where functional annotation databases are curated by third parties and plug in to the *rgsepd* software package. The R code wraps subprocesses for differential expression, set enrichment, and set based projection scoring. The orange cylinder of sample data indicates a normalization produced by DESeq2 with useful expression measurements. Within the Projection Engine box are small diagrams of the integral vector projections and clustering analyses

We built GSEPD as a Bioconductor package named rgsepd to ensure that it is readily available, simple to install, and bundled with both test data and documentation. The system architecture of GSEPD is shown in Fig. 1. The interface to GSEPD is a short list of R commands and all the functions are fully automatic after providing the input data as a matrix. GSEPD generates all tables and figures for the input data within minutes.

GSEPD requires two types of input data to run: the multisample RNA-seq raw counts matrix and sample information matrix. Input should be loaded as a matrix in R with RefSeq ID numbers as row and sample identifiers as column names. The sample information matrix is used to link sample identifiers with test conditions and short labels (for plotting into figures). Given input data, GSEPD automatically computes DE genes between two groups with default parameters of DESeq2, adjusted if necessary for small sample counts [3]. GSEPD also utilizes GOSeq [8] for GO term enrichment analysis, once each for downregulated, upregulated and all genes in the DE gene list.

One of the novel features of GSEPD is to focus on each significantly enriched GO term and assess how samples are segregated with respect to the expression of genes in that GO term. In order to study if samples segregate in their original groups with respect to a particular GO term, GSEPD performs clustering of samples based on the expression of all genes in a significantly enriched GO term. GSEPD can also incorporate non-tested samples (i.e., samples that are not in the predefined groups) in clustering to enable investigators label unclassified or indeterminate samples by their expression profiles among GO terms relevant to the experiment.

GO term-based clustering of samples is performed by using k-means clustering where *k* = 2. Briefly, for a given GO term with *N* genes, each sample is represented as an *N*-dimensional vector of expression of all genes in the GO term. To avoid broad GO terms associated with thousands of genes, only GO terms with less than *m* (*m* = 31 by default) genes are evaluated by GSEPD for clustering.

To assess the quality of the clustering outcome, a validity score called V-measure [16] is computed. The V-measure computes the concordance between cluster assignments and actual class labels of the samples. The V-measure of a clustering is the harmonic mean of the cluster’s *homogeneity* and *completeness*. A cluster’s homogeneity is computed based on the entropy of class labels within the cluster, i.e., maximum homogeneity is achieved when all members of the cluster belongs to the same cluster. The completeness of a cluster is computed based on what percent of members of a class are assigned to the cluster. A cluster would have maximum completeness if it has all members of a class. In ideal cases, clusters should be homogenous and complete.

In order to assess the significance of V-measure score, GSEPD computes an empirical *p*-value for each GO term-based clustering by permuting sample group labels (i.e., class labels) and re-calculating the V-measure. The *p*-value is the proportion of random assignments that achieve a higher V-measure. By default, GSEPD performs adaptive permutation up to 400 times to resolve segregation by *p* < 0.01.

GSEPD visualizes significant GO terms in scatter plots and subspace principle component analysis (PCA) figures to allow further exploration of the results by the user. Vector projection of samples is performed based on gene set of the GO term to score each sample’s similarity to the centroid of each group and to highlight any outlier samples for the gene set.

**Fig. 2:**
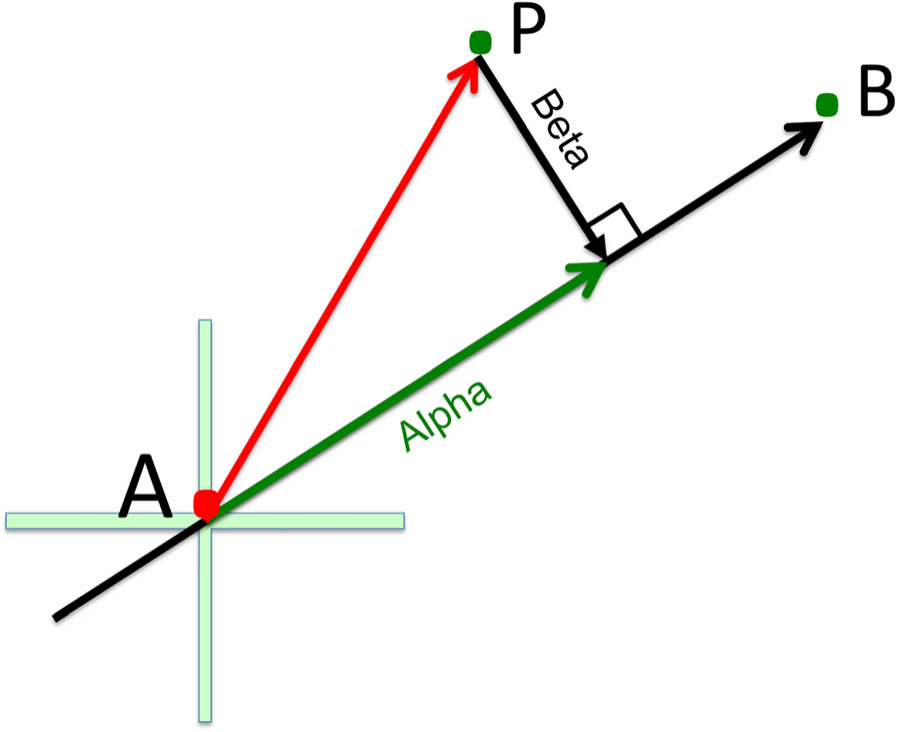
Vector Projection Illustration. With the origin at the cross, vector AP is projected onto vector AB, yielding the green projection. In GSEPD, the point A is the centroid of class A, and point B is the centroid of class B. Point P is any one sample. The green vector is the alpha score and the black perpendicular line from point P is the beta score

In order to assess the concordance between group label of a sample and its localization in the clustering, GSEPD performs vector projection. First, we define the mean expression of the GO term gene set in samples of each group as the centroid of the group, and define an axis connecting both group centroids where one of the centroids is chosen as the origin in a N-dimensional Euclidean space (Fig. 2). Each sample is projected on this axis to compute two scores named *alpha* and *beta*. The alpha score is the distance between projected point on the axis to the origin and the beta score is the Euclidean distance between the sample and the projected point in the axis (Fig. 2). Beta score measures the goodness of fit and flag samples which do not fit the linear assumptions of the two-group comparison performed by DESeq2 whereas alpha score is used to measure the confidence of the cluster assignments. Alpha and beta scores are computed for samples from other groups and can help assess how samples from other groups “behave” for a given GO term.

GO term-based clustering and vector projection is performed for each significant GO term with gene sets ≤ *m*, creating an alpha and beta score for each sample and GO term pair. GSEPD produces heatmaps of gene expression for DE genes, heatmaps of alpha scores for significant GO terms, multi-panel scatterplots of genes in significant GO terms, PCA plots of samples and tables. All thresholds and parameters are configurable before runtime, and configurable output folders and formulaic file naming conventions ensure easy reproducibility or automated parameter sweeps. A tutorial and explanation of all outputs are available within the package vignette/manuals.

## Results and discussion

We run GSEPD on a time series dataset (five time points with two replicates) along the differentiation of H1ESC cells into cardiomyocytes (NCBI SRA accession number SRP048993) [15]. We used GSEPD to compare samples of day 3 and 5, which is a critical turning point between early tissue development and heart muscle precursors [15]. Pairwise comparison of all time points revealed that time points day 3 and day 5 had the fewest DE genes (3279 genes with *p* < 0.05, comprising 2214 GO terms with p < 0.05, 1073 of which were found to cluster samples with a significant V-measure score *p* < 0.01).

**Fig. 3:**
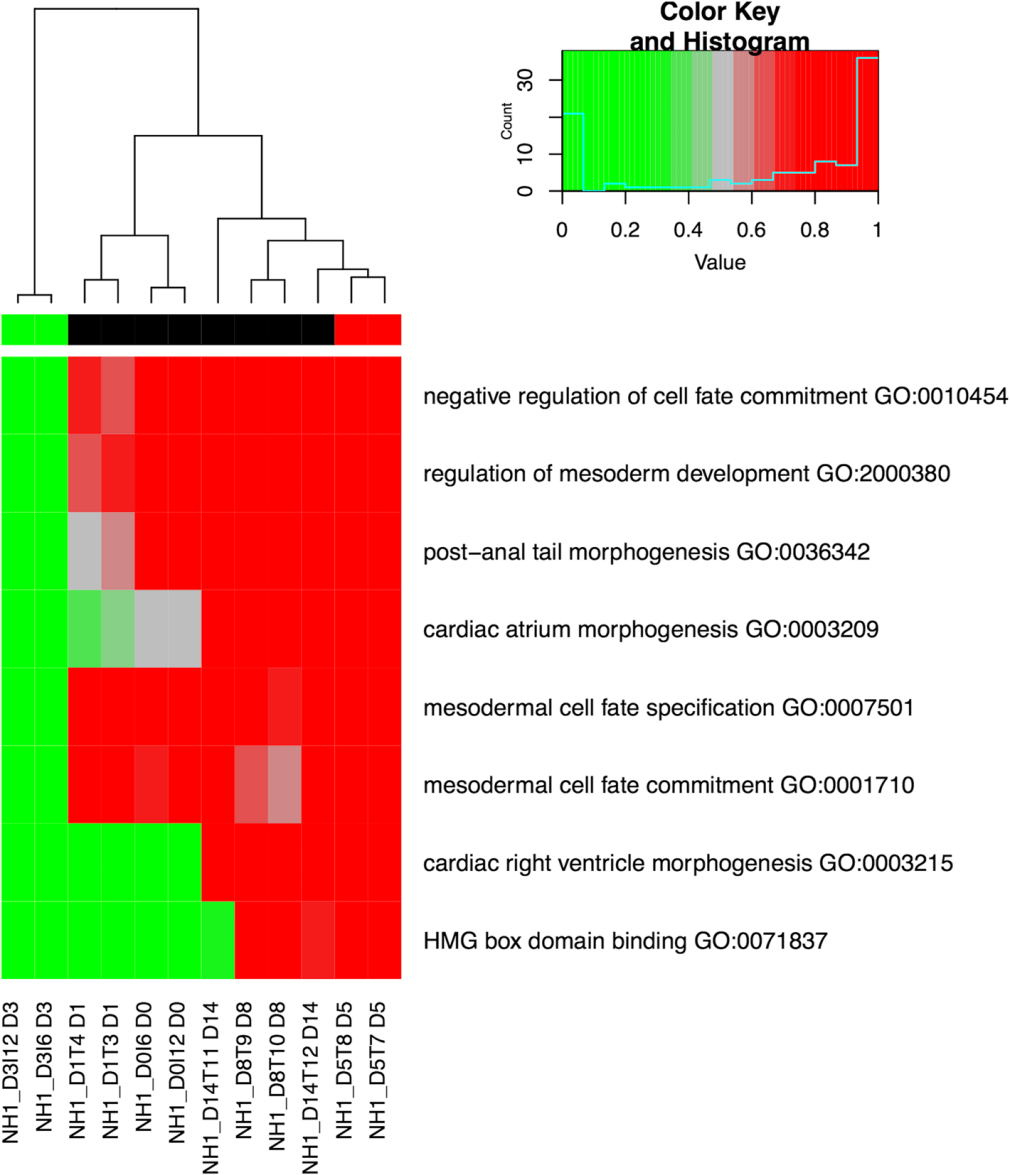
GSEPD Results from the H1ESC Study. The H1ESC dataset is evaluated with GSEPD’s Alpha/Beta scores. Notes along the bottom are a coded sample identifier ending in the time point name D3 for day 3, D1 for day 1, and so on. This figure shows GO terms with significant segregation between day 3 (green) and day 5 (red). GSEPD was instructed via input parameter to display only the top 8 results. The color bar across the top indicates which samples were part of the DESeq2 contrast, here day 3 in green versus day 5 in red, with black denoting non-tested samples

The heatmap of alpha scores (HMA) plot is shown in Fig. 3. The HMA plot can visualize if any sample “behave” similar to its own group or some other group. For instance, for the GO term “cardiac atrium morphogenesis,” the day 3 samples are unique (i.e., bright green), the day 0 and the day 1 samples have average alpha scores (i.e., faded gray) with the day 1 samples are slightly more similar to the day 3 samples, while the samples from later days (i.e., day 8 and 14) behave quite similar to the day 5 samples.

The results in the HMA plot also show that the day 3 samples were unique in GO terms “mesodermal cell fate specification”, “mesodermal cell fate commitment”, “negative regulation of cell fate commitment”, and “regulation of mesoderm development,” suggesting a unique spike of gene activation that deactivated on all other time points. With no biological systems background knowledge, the user of GSEPD can thus extract pathway activation knowledge from RNA-seq count data.

**Fig. 4:**
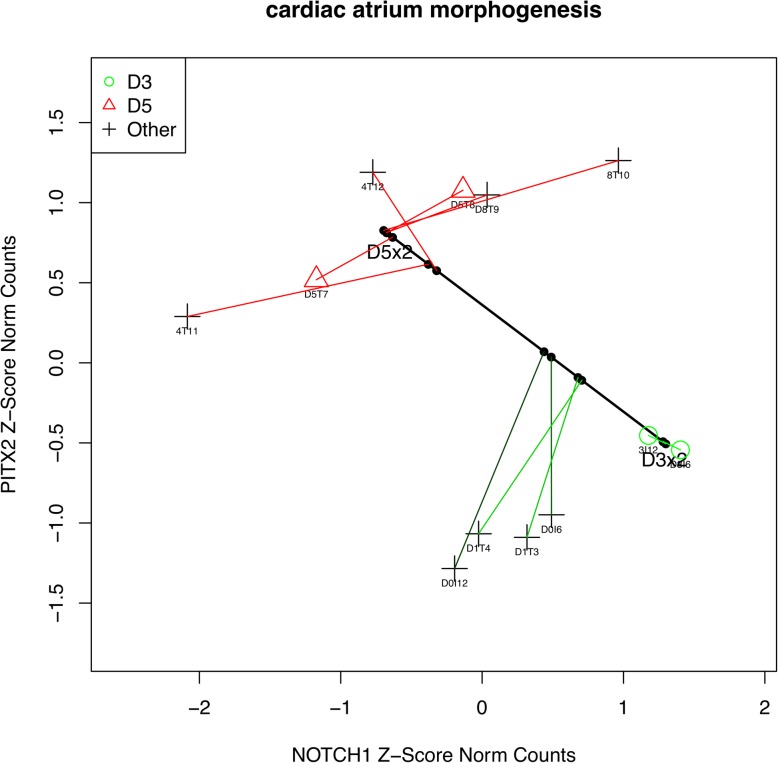
Scatterplot of Two Genes. Corresponding to ‘atrial cardiac muscle tissue development’ GO term in Fig. 3, this diagram is one part of generated file GSEPD.D3x2.D5x2.GO0003209.pdf (first two genes). Points as triangles, circles, and crosses correspond to the input samples. Solid dots indicate the projection coordinate. Labels D5x2 and D3x2 indicate class centroids of the comparison of two samples of day 5 versus two samples of day 3. The small point labels are specified by the user as each sample’s “shortname,” a parameter given to GSEPD

GSEPD also extracts significant GO terms into multi-page scatterplots of genes showing orthogonal views of samples on the high-dimensional clusters. For instance, for the “cardiac atrium morphogenesis” a 28-gene GO term in the HMA figure (Fig. 3), a sample scatterplot between *PITX2* and *NOTCH1* is shown in Fig. 4. In this scatterplot *PITX2* is shown downregulated in class day 3 (green) versus class day 5 (red), whereas *NOTCH1* is upregulated by 1.5 units of logged normalized counts. Colored lines (corresponding to cells of the heatmap in Fig. 3) are perpendicular to the thick black axis in the 28-dimensional space (although they do not appear perpendicular in the two-gene subspace), indicating samples of day 0 and day 1 fall between the clusters of the day 3 and the day 5 samples and whereas the day 8 and the day 14 samples are clustered with the day 5 samples for this GO term.

## Conclusions

GSEPD is a user-friendly RNA-seq analysis toolkit. To enable rapid and simple installation and ensure reproducibility of results, GSEPD was implemented as an open source Bioconductor package. By utilizing the GO hierarchy through GOSeq, GSEPD can quickly identify significantly enriched GO terms with DE genes computed by DESeq2. Furthermore, GSEPD can visualize how each sample behaves with regard to each significant GO term. Byproducts including sample PCA figures save time and effort and can identify sample batch effects that may confound analyses and be obscured by rudimentary differential expression produced by other pipelines.

## Availability and requirements

GSEPD is implemented as a Bioconductor package named rgsepd and freely available under GPL-3 license for academic and non-academic usage. The Bioconductor system will install required additional packages DESeq2, GOSeq, and the GO databases, available to any Mac, Linux, and Windows PC. Generating the input matrix will require other tools. Description of the 13 types of figures and 12 types of tables generated by each comparison run are available in the bundled package manuals. Instructions, manuals, and sample data are available in the online help files and the project website at https://bioconductor.org/packages/release/bioc/html/rgsepd.html.

## Abbreviations

DE: Differential expressed
GO: Gene Ontology
HMA: Heatmap of alpha scores
mRNA: messenger RNA
PCA: Principal Component Analysis

## References

[1] Wang Z, Gerstein M, Snyder M (2008). RNA-Seq: a revolutionary tool for transcriptomics. Nat Rev Genet.

[2] Garber M, Grabherr MG, Guttman M, Trapnell C (2011). Computational methods for transcriptome annotation and quantification using RNA-seq. Nat Methods.

[3] Love MI, Huber W, Anders S (2014). Moderated estimation of fold change and dispersion for RNA-seq data with DESeq2. Genome Biol.

[4] Grabherr MG, Haas BJ, Yassour M, Levin JZ, Thompson DA, Amit I, Adiconis X, Fan L, Raychowdhury R, Zeng Q, Chen Z, Mauceli E, Hacohen N, Gnirke A, Rhind N, di Palma F, Birren BW, Nusbaum C, Lindblad-Toh K, Friedman N, Regev A (2011). Full-length transcriptome assembly from RNA-Seq data without a reference genome. Nat Biotechnol.

[5] Schulz MH, Zerbino DR, Vingron M, Birney E (2012). Oases: robust de novo RNA-seq assembly across the dynamic range of expression levels. Bioinformatics.

[6] Trapnell C, Williams BA, Pertea G, Mortazavi A, Kwan G, van Baren MJ, Salzberg SL, Wold BJ, Pachter L (2010). Transcript assembly and quantification by RNA-Seq reveals unannotated transcripts and isoform switching during cell differentiation. Nat Biotechnol.

[7] Williams CR, Baccarella A, Parrish JZ, Kim CC (2017). Empirical assessment of analysis workflows for differential expression analysis of human samples using RNA-Seq. BMC Bioinformatics.

[8] Young MD, Wakefield MJ, Smyth GK, Oshlack A (2010). Gene ontology analysis for RNA-seq: accounting for selection bias. Genome Biol.

[9] Kim D, Pertea G, Trapnell C, Pimentel H, Kelley R, Salzberg SL (2013). TopHat2: accurate alignment of transcriptomes in the presence of insertions, deletions and gene fusions. Genome Biol.

[10] Dobin A, Davis CA, Schlesinger F, Drenkow J, Zaleski C, Jha S, Batut P, Chaisson M, Gingeras TR (2013). STAR: ultrafast universal RNA-seq aligner. Bioinformatics.

[11] Robinson MD, McCarthy DJ, Smyth GK (2010). edgeR: a Bioconductor package for differential expression analysis of digital gene expression data. Bioinformatics.

[12] Ritchie ME, Phipson B, Wu D, Hu Y, Law CW, Shi W, Smyth GK (2015). Limma powers differential expression analyses for RNA-sequencing and microarray studies. Nucleic Acids Res.

[13] Wang X, Cairns MJ (2014). SeqGSEA: a Bioconductor package for gene set enrichment analysis of RNA-Seq data integrating differential expression and splicing. Bioinformatics.

[14] Ramsköld D, Wang ET, Burge CB, Sandberg R (2009). An abundance of ubiquitously expressed genes revealed by tissue transcriptome sequence data. PLoS Comput Biol.

[15] Kim MS, Horst A, Blinka S, Stamm K, Mahnke D, Schuman J, Gundry R, Tomita-Mitchell A, Lough J (2015). Activin-a and Bmp4 levels modulate cell type specification during CHIR-induced cardiomyogenesis. PLoS One.

[16] A. Rosenberg and J. Hirschberg, "V-Measure: A Conditional Entropy-Based External Cluster Evaluation Measure", *in EMNLP-CoNLL*, 2007, 7, no., p. . 410–420"V-Measure: A Conditional Entropy-Based External Cluster Evaluation Measure", *in EMNLP-CoNLL*, 7, no., 2007, p. . 410–420.

